# Mavacamten has a differential impact on force generation in myofibrils from rabbit psoas and human cardiac muscle

**DOI:** 10.1085/jgp.202012789

**Published:** 2021-04-23

**Authors:** Beatrice Scellini, Nicoletta Piroddi, Marica Dente, Giulia Vitale, Josè Manuel Pioner, Raffaele Coppini, Cecilia Ferrantini, Corrado Poggesi, Chiara Tesi

**Affiliations:** 1 Department of Experimental and Clinical Medicine, Division of Physiology, University of Florence, Florence, Italy; 2 Department of Neuroscience, Psychology, Drug Sciences, and Child Health, University of Florence, Florence, Italy

## Abstract

Mavacamten (MYK-461) is a small-molecule allosteric inhibitor of sarcomeric myosins being used in preclinical/clinical trials for hypertrophic cardiomyopathy treatment. A better understanding of its impact on force generation in intact or skinned striated muscle preparations, especially for human cardiac muscle, has been hindered by diffusional barriers. These limitations have been overcome by mechanical experiments using myofibrils subject to perturbations of the contractile environment by sudden solution changes. Here, we characterize the action of mavacamten in human ventricular myofibrils compared with fast skeletal myofibrils from rabbit psoas. Mavacamten had a fast, fully reversible, and dose-dependent negative effect on maximal Ca^2+^-activated isometric force at 15°C, which can be explained by a sudden decrease in the number of heads functionally available for interaction with actin. It also decreased the kinetics of force development in fast skeletal myofibrils, while it had no effect in human ventricular myofibrils. For both myofibril types, the effects of mavacamten were independent from phosphate in the low-concentration range. Mavacamten did not alter force relaxation of fast skeletal myofibrils, but it significantly accelerated the relaxation of human ventricular myofibrils. Lastly, mavacamten had no effect on resting tension but inhibited the ADP-stimulated force in the absence of Ca^2+^. Altogether, these effects outline a motor isoform–specific dependence of the inhibitory effect of mavacamten on force generation, which is mediated by a reduction in the availability of strongly actin-binding heads. Mavacamten may thus alter the interplay between thick and thin filament regulation mechanisms of contraction in association with the widely documented drug effect of stabilizing myosin motor heads into autoinhibited states.

## Teaser

Scellini et al. show that mavacamten, a preclinical inhibitor of sarcomeric myosins, has a fast and reversible mechanical action on skeletal and cardiac myofibers that is mediated by a shift of motor heads out of a force-generating cycle, with no effect on the kinetics of cardiac force development.

## Introduction

Mavacamten (MAVA), formerly known as MYK-461 (MyoKardia), is an allosteric inhibitor of sarcomeric myosins identified in a small-molecule screening of potential drugs for the treatment of human hypertrophic cardiomyopathy (HCM; Green et al., 2016; Spudich et al., 2016; Alsulami and Marston, 2020). In the last 10 yr, a lot of experimental evidence has supported the hypothesis that HCM could mainly result from an increase in the availability of myosin heads for entering the actomyosin chemomechanical cycle (Spudich, 2014; Garfinkel et al., 2018; Trivedi et al., 2018; Spudich, 2019) associated with a shift from the so-called autoinhibited or super-relaxed (SRX) state (Stewart et al., 2010; Hooijman et al., 2011; Alamo et al., 2017) to a disordered-relaxed (DRX) state (McNamara et al., 2015). The increase in DRX over SRX state proportion would contribute to the observed increase in the energy cost of contraction, triggering a complex signaling cascade leading to overt hypertrophic remodeling and a number of functional modifications (Ashrafian et al., 2003; Vitale et al., 2021).

In this perspective, MAVA has been proved to be an effective agent as it strongly reduces maximal actin-activated myosin ATPase (both myofibrillar and acto-S1) in a dose-dependent manner, with a half-maximal inhibitory concentration (IC_50_) in the submicromolar range for cardiac myosin (Green et al., 2016) and ∼10 times higher for skeletal myosin (Kawas et al., 2017). At the same time, MAVA stabilizes the autoinhibited SRX state in human and bovine purified β-cardiac myosin (Anderson et al., 2018; Rohde et al., 2018) as well as in porcine and mouse cardiac fibers (Anderson et al., 2018; Toepfer et al., 2019) and human cultured cardiomyocytes from induced pluripotent stem cell-derived cardiomyocytes (iPSC-CMs) expressing various HCM-linked β-myosin heavy chain (*MYH7*) variants (Toepfer et al., 2020). When tested on myocardial function, MAVA has been proved to similarly reduce in vitro contractility in control and HCM animals (Green et al., 2016; Anderson et al., 2018; Mamidi et al., 2018) as well as relieve left ventricular outflow tract obstruction in HCM feline hearts (Stern et al., 2016) and suppress the development of the hypertrophic phenotype in HCM mouse models. Recently, it has been shown that MAVA decreases the Ca^2+^ sensitivity in both human and mouse ventricle (Awinda et al., 2020; Awinda et al., 2021) and rescues the increase in Ca^2+^ sensitivity caused by thin-filament HCM mutations in mouse models (Sparrow et al., 2019). These observations, indicating a window of opportunities in which restoration of physiological sarcomere performance may prevent cardiac remodeling, are the basis for the present use of MAVA in preclinical/clinical trials for HCM treatment, as confirmed by the very promising results of a phase 2 open-label trials (Heitner et al., 2019) and a recently completed multicenter placebo-controlled randomized phase 3 trial (EXPLORER-HCM; Olivotto et al., 2020).

Despite the advancing of clinical studies, very little is known of MAVA action on the mechanics of contraction of fast skeletal and slow/cardiac muscles. The study of MAVA effects in human cardiac tissue has only very recently started to go beyond a very preliminary stage with direct measurements of the drug action at physiological temperature (37°C) in control permeabilized human left ventricular strips at different Ca^2+^ activation levels and sarcomere lengths (Awinda et al., 2020). Results of this work showed that low doses of MAVA decrease maximal force development and work production as well as Ca^2+^ sensitivity of human myocardium but preserve the length-dependent activation mechanism.

In the present work, we investigate MAVA action as a cross-bridge cycle inhibitor and potential modulator of thick/thin filament–mediated regulation of contraction with a systematic study of its sarcomeric impact both in human ventricular and rabbit psoas myofibrils. This comparative approach is of interest because MAVA, besides its intrinsic interest for clinical studies, could also become a tool to unravel key differences between the mechanochemical cycle and the regulation mechanisms of muscles expressing fast and slow motor (and other associated sarcomeric protein) isoforms. In addition, the rabbit psoas model represents the “gold standard” of biochemical and mechanical studies of muscle contraction and offers a comparative tool for investigating less known and challenging experimental models such as the human myocardium. In this comparative study, we use the best-suited preparation to investigate the effects of ligands and sudden changes in their concentrations with high time resolution, i.e., single myofibrils mounted in isometric conditions and subjected to rapid solution changes (Tesi et al., 2000).

To this aim, we tested the effects of MAVA on force generation of slow and fast striated muscle using thin bundles of myofibrils from frozen samples of the left ventricle of human donors mainly expressing *MYH7 *(≥95%; Reiser et al., 2001) or fast rabbit skeletal muscle mainly expressing fast skeletal muscle myosin *MYH1* (≥96%; Aigner et al., 1993). We demonstrate that in both myofibril systems, the action of MAVA on myofibril Ca^2+^-activated force is fast and fully reversible. MAVA induces an immediate shift of cross-bridges toward detached states, leading to force decrease and fast/slow muscle type–specific changes in the kinetics of force generation and relaxation. Furthermore, in the conditions of the study, MAVA had no effects on the resting properties of fast skeletal and human ventricular muscle, while it was found to inhibit ADP–stimulated force generation in the absence of Ca^2+^. Analysis of the kinetics of force recovery following sudden MAVA removal from the myofibrils supports the known effect of MAVA in shifting detached myosin heads toward a sequestered SRX state.

## Materials and methods

### Preparation of myofibrils from rabbit fast skeletal and human cardiac samples

Single myofibrils or thin bundles of myofibrils were isolated from fast psoas muscle of rabbit killed by pentobarbital administration (120 mg/kg) through the marginal ear vein according to the procedure established by the European Union Council on the Use of Laboratory Animals (Directive 2010/63/EU) and using protocols approved by the Ethics Committee for Animal Experiments of the University of Florence. After dissection, muscles were cut in strips ∼0.5 cm wide, tied at rest length to rigid wood sticks, and stored at −20°C for no more than 6 mo in a 200 mM ionic strength rigor solution (100 mM KCl, 2 mM MgCl_2_, 1 mM EGTA, and 50 mM Tris, pH 7.0) supplemented with glycerol 50%. Single myofibrils or bundles of two or three myofibrils were prepared by homogenization of glycerinated psoas muscle as previously described (Tesi et al., 2000).

Myofibrils from human cardiac muscle were prepared by homogenization of frozen healthy donor human interventricular heart septum samples. The frozen samples were stored at −80°C at the da Vinci Biobank of the University of Florence. Experiments involving the use of human samples had been approved by the local ethics committee (Azienda Ospedaliera Universitaria Careggi; protocol no. 2006/0024713−28/06/2006, renewed 10/2009).

Thin strips dissected from the interventricular septum sample were permeabilized overnight in ice-cold relaxing solution added with 1% Triton-X 100. Demembranated strips were then homogenized in relaxing solution to produce myofibril suspensions (Piroddi et al., 2007; Belus et al., 2008).

Rabbit fast skeletal and human cardiac myofibril suspensions, stored at 0–4°C, were stable and were used for up to 5 d. All solutions to which the myofibrils were exposed contained a cocktail of protease inhibitors including leupeptin (10 µM), pepstatin (5 µM), phenylmethylsulphonyl fluoride (200 µM), and E64 (10 µM), as well as NaN_3_ (500 µM) and 500 µM dithiothreitol. [Ca^2+^] in experimental solutions was expressed as pCa = −log[Ca^2+^].

### Myofibril experiments

Bundles of few myofibrils (skeletal: 40–80 µm long and 1–3 µm wide; human cardiac: 25–70 µm long and 2–5 µm wide) were mounted in a force recording apparatus as previously described (Colomo et al., 1998; Tesi et al., 2000). Briefly, myofibrils were mounted horizontally between two glass microtools in a temperature-controlled chamber (15°C) filled with relaxing solution (pCa 9.0). One tool was connected to a length-control motor that could produce rapid (<1 ms) length changes. The second tool was a calibrated cantilever force probe (2–6 nm/nN; frequency response 2–5 kHz). Force was measured from the deflection of the image of the force probe projected on a split photodiode. The initial sarcomere length of the preparations was set just above the slack length. Myofibrils were activated and relaxed in control (Ctrl) conditions by rapidly translating the interface between two flowing streams of activating (pCa 4.5) and relaxing (pCa 9.0) solutions across the preparation. The solution change was complete in <5 ms (Colomo et al., 1998). Maximal isometric developed force was measured after normalization for the cross-sectional area of the preparation (*P*_*0*_). The rate of force development (*k*_ACT_) and the rate of force redevelopment following a release–restretch protocol (*k*_TR_; Brenner, 1988) were estimated from the time required to reach 50% of *P*_*0*_.

The rate constant of the early slow force decline (slow *k*_REL_) was estimated from the slope of the regression line fitted to the tension trace normalized to tension just before relaxation. The early slow force decay (linear phase of relaxation) is assumed to be the initial part of an exponential process that, if it lasted for the whole relaxation transient, would lead force to its final steady-state value with a rate constant equal to the initial slope of force decay divided by the amplitude of the overall force decay. The duration of the slow relaxation phase was estimated from the start of the solution change signal. Experimental traces were not used to measure slow *k*_REL_ when the mechanical artifacts produced by the solution change did not allow reproducible measurements (±10%) by two different investigators. The rate constant for the final fast phase of tension decline (fast *k*_REL_) was estimated from a monoexponential fit (Tesi et al., 2002b; Poggesi et al., 2005). The same mechanical measurements were performed in the presence of selected [MAVA] in both relaxing and activating solutions (P_MAVA_, tension in the presence of [MAVA]).

Resting tension at pCa 9.0 was measured by imposing large length releases to the myofibrils mounted at their initial length for force recording; myofibrils were restretched back to their initial length after recording the zero-force level. Sarcomere length–resting tension relationships were obtained with the same procedure by measuring sarcomere lengths and resting tensions at different increasing initial myofibril lengths. In the MAVA jump experiments both channels of the perfusing pipette were loaded with activating solutions, one without MAVA (Ctrl) and the other one added with MAVA. Myofibrils were activated by translating the interface between the relaxing solution in the experimental chamber and the Ctrl activating solution. Once a steady plateau of isometric force was attained, the perfusing flow was rapidly switched to the activating solution containing selected concentrations of MAVA and back. Force transients resulting from exposure and removal of MAVA were then recorded, and their rates (named *k*_MAVA+_ and *k*_MAVA−_, respectively) were estimated from the observed half-time of force changes.

### Solutions for mechanical experiments

All activating and relaxing solutions were calculated as described previously (Tesi et al., 2000) at pH 7.0 and contained 10 or 1 mM of total EGTA (CaEGTA/EGTA ratio set to obtain different pCa values in the range of 9.00–4.50), 5 mM MgATP, 1 mM free Mg^2+^, 10 mM 3-(N-morpholino) propane sulfonic acid, propionate, and sulfate to adjust the final solution to an ionic strength of 200 mM and a monovalent cation concentration of 155 mM. Creatine phosphate (10 mM) and creatine kinase (200 U/ml^−1^) were added to all solutions to minimize alterations in the concentration of MgATP and its hydrolysis products. Creatin kinase and creatine phosphate were not present in solutions containing 5 mM MgADP. In some cases, contaminant [P_i_] (∼170 µM in standard solutions) was reduced to <5 µM (P_i_-free solutions) by a P_i_-scavenging enzyme system (purine-nucleoside-phosphorylase with substrate 7-methyl-guanosine; Tesi et al., 2002b). MAVA (Axon Medchem) was dissolved in DMSO to give a 10 mM stock solution. This solution was mixed with relaxing and activating solutions to test final 0.1–50 µM [MAVA] corresponding to 0.001–0.5% DMSO (vol/vol). In Ctrl conditions, all solutions were normalized for DMSO content. All chemicals and enzymes were purchased from Sigma-Aldrich (Merck Life Science).

### Data acquisition and analysis

Force and length signals were continuously monitored throughout the experiments using commercial software and programs (LabVIEW; National Instruments) modified for our use. The same signals were also recorded during experimental protocols and later used for data analysis. Data measurements were made directly with commercial software (Origin; OriginLab) and an in-house-written LabVIEW analysis program that converted the analogic signals to numeric values. The data are expressed and plotted as the mean ± SEM obtained from *n* myofibrils. Comparisons were performed by two-tailed Student’s *t* test. Differences between groups were considered statistically significant when P ≤ 0.05. One-way ANOVA with a Tukey post-hoc test was used to compare multiple myofibril groups (and their mechanical parameters) in the presence of different [P_i_].

## Results

### MAVA has a fast and fully reversible effect on maximal Ca^2+^-activated force with different selectivity in fast skeletal and human cardiac myofibrils

**Figure 1.**
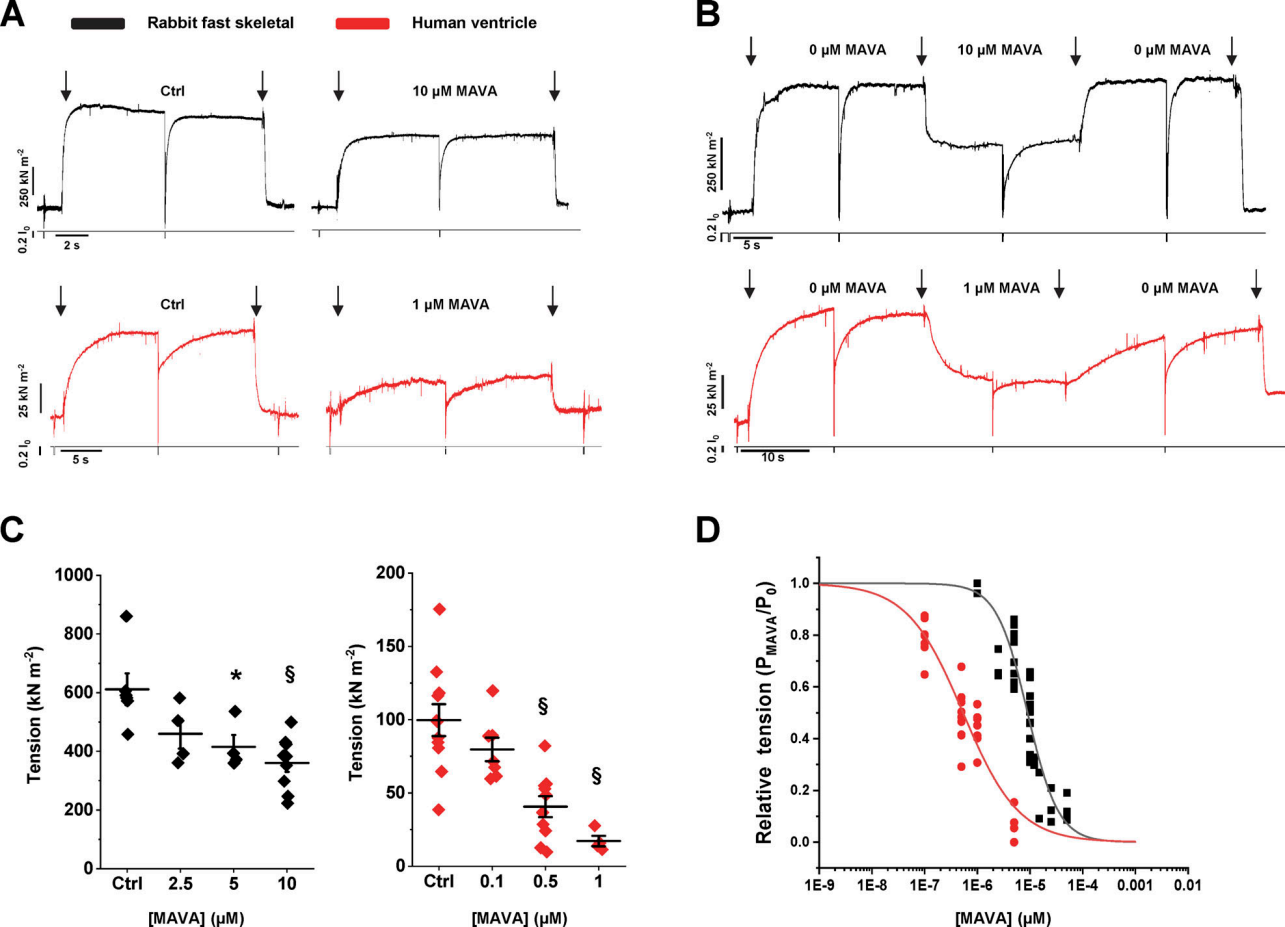
**MAVA decreases maximal Ca^2+^-activated force of skeletal and cardiac myofibrils.** **(A)** Representative contraction–relaxation cycle of rabbit fast skeletal (black) and human ventricular (red) myofibrils that are maximally Ca^2+^-activated (first arrow) and fully relaxed (second arrow) by fast solution switching (15°C) in the absence (left) and presence (right) of 10 µM and 1 µM MAVA. Lower traces represent length changes (20–40% of the initial length) applied in relaxing solution to assess passive tension and during steady Ca^2+^-activation for *k*_TR_ evaluation. **(B)** Representative MAVA-jump traces from fast skeletal (black) and human ventricular (red) myofibrils. Arrows mark the fast solution changes as indicated. **(C)** Mean data of maximal tension in pCa 4.5 for fast skeletal (black) and human ventricle (red) myofibrils from contraction–relaxation cycles like those in A. *, P < 0.05; §, P < 0.001 versus Ctrl. Error bars, ± SEM. **(D)** Relationship between different MAVA concentration and corresponding relative tension levels at pCa 4.5 obtained during the jump protocols in skeletal (black) and cardiac (red) myofibrils. P_0_, Ctrl tension; P_MAVA_, tension in the presence of correspondent [MAVA].

Two different experimental protocols were used to study the impact of MAVA on maximal Ca^2+^-activated force of thin bundles of myofibrils (1.5–5.0 µm diameter) isolated from fast skeletal or human ventricular muscle. Both myofibril types were mounted at 15°C and optimal sarcomere length in relaxing solution (pCa 9.0) and then fully activated (pCa 4.5) by rapid solution switching. Unlike otherwise specified, all solutions had a contaminant [P_i_] of ∼170 μM (Tesi et al., 2000) and were normalized for DMSO used as a solvent for MAVA. In the first protocol, we compared maximal isometric force measured in contraction–relaxation cycles of two different batches of myofibrils, one tested in Ctrl conditions and the other one in the presence of relaxing and activating solutions added with selected concentrations of MAVA (Fig. 1 A; Table 1). The drug concentration in the myofibril lattice was assumed to be equal to that of the perfusing solution continuously flowing through the preparation. In the second protocol, the effect of MAVA on isometric force was measured in the same myofibril from the amplitude of a “MAVA jump” (see Materials and methods; i.e., first activating the myofibril in Ctrl conditions, and once a steady plateau of isometric force was attained, switching to an activating solution containing a given MAVA concentration and then back to the Ctrl solution; Fig. 1 B). Resting sarcomere lengths of rabbit psoas and human ventricular myofibrils were 2.77 ± 0.01 µm (*n* = 82) and 2.14 ± 0.01 µm (*n* = 73), respectively.

**Table 1.** Effect of MAVA on the mechanical properties of myofibrils from rabbit fast skeletal and human cardiac ventricle

Myofibril batch	Tension generation			Relaxation in slow or fast phase		
	P_0_	*k*_ACT_	*k*_TR_	D_slow_	Slow *k*_REL_	Fast *k*_REL_
	kN m^−2^	s^−1^	s^−1^	ms	s^−1^	s^−1^
**Rabbit fast skeletal**						
0 µM MAVA	612 ± 54 (6)	7.28 ± 0.51 (6)	7.61 ± 0.53 (6)	88 ± 7 (6)	2.09 ± 0.16 (6)	41 ± 5.6 (6)
2.5 µM MAVA	460 ± 51 (4)	4.13 ± 0.61^a^ (4)	4.58 ± 0.32^a^ (4)	76 ± 6 (4)	1.82 ± 0.25 (4)	37 ± 3.4 (4)
5 µM MAVA	415 ± 41^b^ (4)	2.35 ± 0.08^c^ (4)	3.02 ± 0.20^c^ (4)	74 ± 8 (4)	1.40 ± 0.35 (4)	30 ± 1.6 (4)
10 µM MAVA	360 ± 30^d^ (9)	3.05 ± 0.12^c^ (9)	3.36 ± 0.27^c^ (9)	82 ± 6 (9)	1.54 ± 0.20 (9)	36 ± 2.6 (9)
**Human ventricle**						
0 µM MAVA	100 ± 11 (11)	0.68 ± 0.04 (11)	0.53 ± 0.02 (11)	176 ± 16 (9)	0.40 ± 0.05 (10)	4.25 ± 0.41 (9)
0.1 µM MAVA	80 ± 8 (7)	0.64 ± 0.04 (6)	0.55 ± 0.03 (7)	130 ± 10 (6)	0.37 ± 0.07 (6)	6.35 ± 0.66^b^ (7)
0.5 µM MAVA	41 ± 7^d^ (10)	0.60 ± 0.04 (10)	0.56 ± 0.04 (10)	152 ± 17 (10)	0.77 ± 0.11^a^ (9)	5.97 ± 0.46^b^ (10)
1 µM MAVA	17 ± 4^d^ (4)	0.68 ± 0.02 (4)	0.75 ± 0.05^d^ (3)	200 ± 37 (4)	0.86 ± 0.17^a^ (4)	4.94 ± 0.53 (4)

Each group of data was collected in different myofibril batches activated and relaxed by fast solution switch. Rabbit fast skeletal and human ventricle myofibrils were treated with different concentrations of MAVA as described. All values are given as mean ± SEM; values in parentheses are the myofibril numbers. D_slow_, duration of the slow phase of relaxation.

a P < 0.005 versus Ctrl myofibrils (Student’s *t* test).

b P < 0.05 versus Ctrl myofibrils (Student’s *t* test).

c P < 0.0001 versus Ctrl myofibrils (Student’s *t* test).

d P < 0.001 versus Ctrl myofibrils (Student’s *t* test).

The jump protocol provides an internal control for estimating with high resolution the impact on force of any perturbation in ligand concentration inside the sarcomeric lattice (Tesi et al., 2000).

Mean maximum isometric tension of all myofibrils tested with the two protocols in Ctrl conditions (in the presence of DMSO) was 462 ± 27 (*n* = 29) and 99 ± 8 (*n* = 58) kN m^−2^, for rabbit psoas and human ventricular myofibrils, respectively. These values are similar to those previously reported from the same myofibril system at 15°C in the absence of DMSO (Tesi et al., 2000; Piroddi et al., 2007). These results, as well as the lack of any significant effect observed in direct test of DMSO up to 0.5% on skeletal and cardiac myofibril mechanics (P = 0.99; *n* = 15), confirm previous observations reporting no significant effect of DMSO on the contractile behavior of striated muscle (McCormick et al., 2010).

As shown in Table 1 and Fig. 1, C and D, MAVA had a strong inhibitory effect on maximal Ca^2+^-activated tension with a sensitivity to the drug that was approximately one order of magnitude higher in human ventricular myofibrils (IC_50_ 0.58 ± 0.07 µM) than in rabbit psoas myofibrils (IC_50_ 7.00 ± 0.90 µM). These IC_50_ values are in the same range of concentrations of those previously reported for half-maximal–activated ATPase of the same myofibril or actomyosin systems (Kawas et al., 2017; pCa 6.00) as well as for the ATPase of mouse cardiac myofibrils (expressing α-myosin, *MYH6*) at full calcium activation (Green et al., 2016). Interestingly, the effect of MAVA on maximal isometric tension, as observed from MAVA-jump experiments, was found to be extremely fast in both myofibril systems (see below for a description of the kinetics of MAVA force transients) and fully reversible.

**Figure 2.**
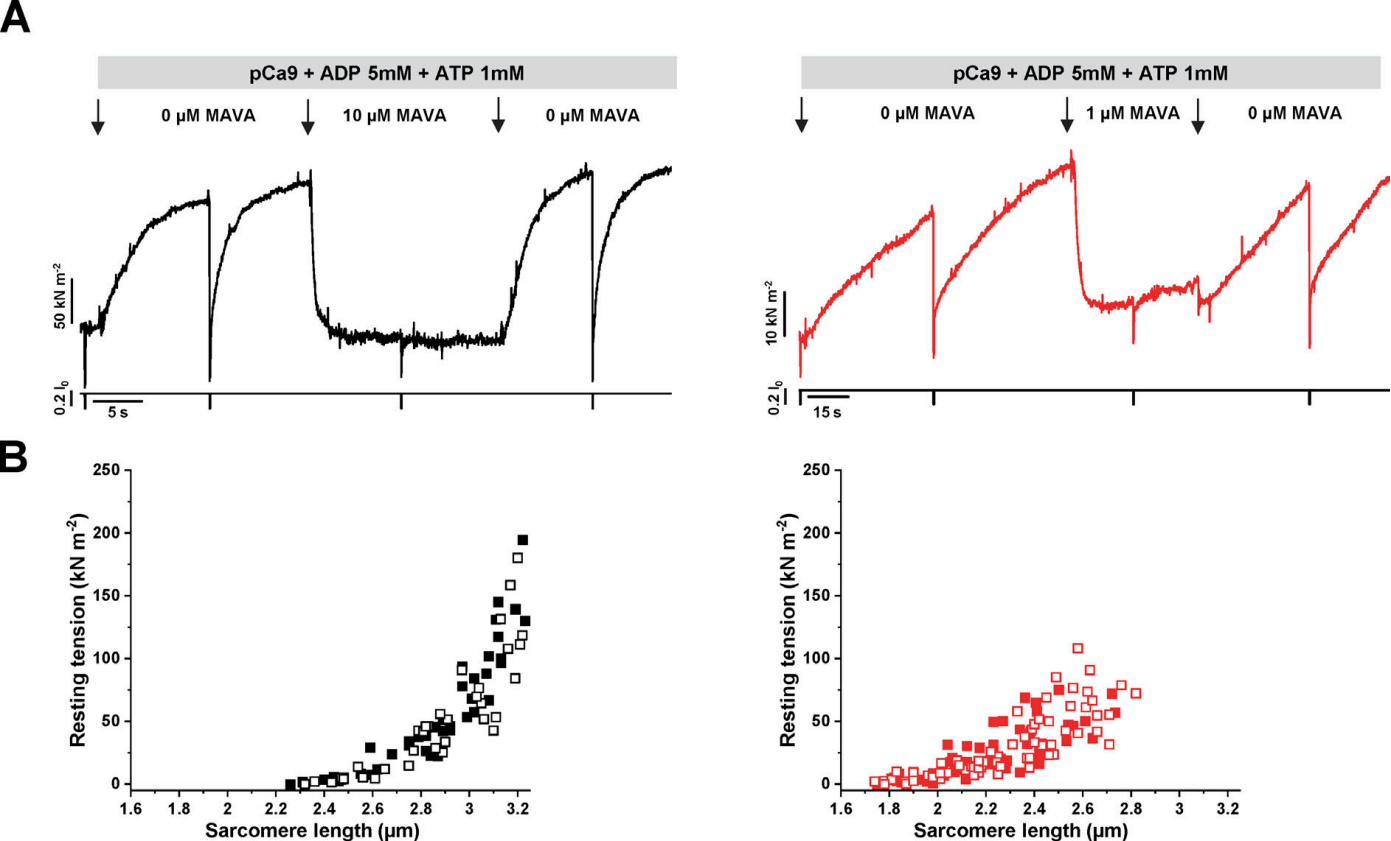
**MAVA inhibits ADP-stimulated contractions in the absence of Ca**^**2+**^**without affecting the sarcomere length–resting tension relationship of rabbit fast skeletal and human ventricle myofibrils. (A)** Representative MAVA-jump traces from Ca^2+^-independent ADP-stimulated contractions of rabbit fast skeletal (black; 10 µM MAVA) and human ventricular (red; 1 µM MAVA) myofibrils. Bars and arrows mark the fast solution changes as indicated. *k*_TR_, 0.6 s^−1^ (rabbit psoas) and 0.04 s^−1^ (human ventricle). **(B)** Sarcomere length–resting tension relationships of rabbit psoas (black symbol; *n* = 8) and human cardiac ventricle (red symbol; *n* = 13) myofibrils in Ctrl relax conditions (filled symbols) or the presence of MAVA (open symbols). MAVA concentration in relaxing solution was 10 µM for fast skeletal and 1 µM for cardiac ventricle myofibrils. Resting tension and sarcomere length were measured under quasi steady-state conditions (i.e., well after the end of elongation, when most of stress relaxation was over).

Besides the impact on myofibril Ca^2+^-activated contractions, MAVA had also a strong inhibitory effect on Ca^2+^-independent ADP-stimulated contractions (Fig. 2 A). Using relaxing solution (pCa 9.0) with reduced MgATP (1 mM) added with 5 mM MgADP, both rabbit psoas (*n* = 7) and human ventricular myofibrils (*n* = 6) developed a significant amount of tension (67 ± 14 kN m^−2^ and 39 ± 7 kN m^−2^, respectively). When MAVA jumps were performed on these ADP-stimulated contractions at concentrations just above the IC_50_ of the two myofibril systems (10 and 1 µM, respectively), ADP-dependent tension was reduced to values not significantly different from the resting tension measured in Ctrl relaxing solution (0.96 ± 0.06 and 1.04 ± 0.03 for rabbit psoas and human ventricular myofibrils, respectively).

As shown in Fig. 2 B, MAVA had no effect on the passive properties of both rabbit psoas and human ventricular myofibrils as determined by the sarcomere length–resting tension relations measured in Ctrl conditions or in the presence of 1 and 10 µM MAVA, respectively.

### The impact of MAVA on sarcomere force kinetics differs in fast skeletal and human ventricular myofibrils

The rate of isometric force generation was obtained from the time course of the force rise after rapid Ca^2+^activation (*k*_ACT_; Tesi et al., 2000) and/or from the time course of force redevelopment following a release–restretch protocol (*k*_TR_ as in Brenner, 1988). Mean *k*_ACT_ and *k*_TR_ values (in the presence of DMSO) of rabbit psoas and human ventricle myofibrils in activation–relaxation cycles are reported in Table 1. Again, these values are in line with those previously reported from the same myofibril systems at 15°C and confirm the lack of any effect of DMSO on contraction kinetics (Tesi et al., 2000; Piroddi et al., 2007).

**Figure 3.**
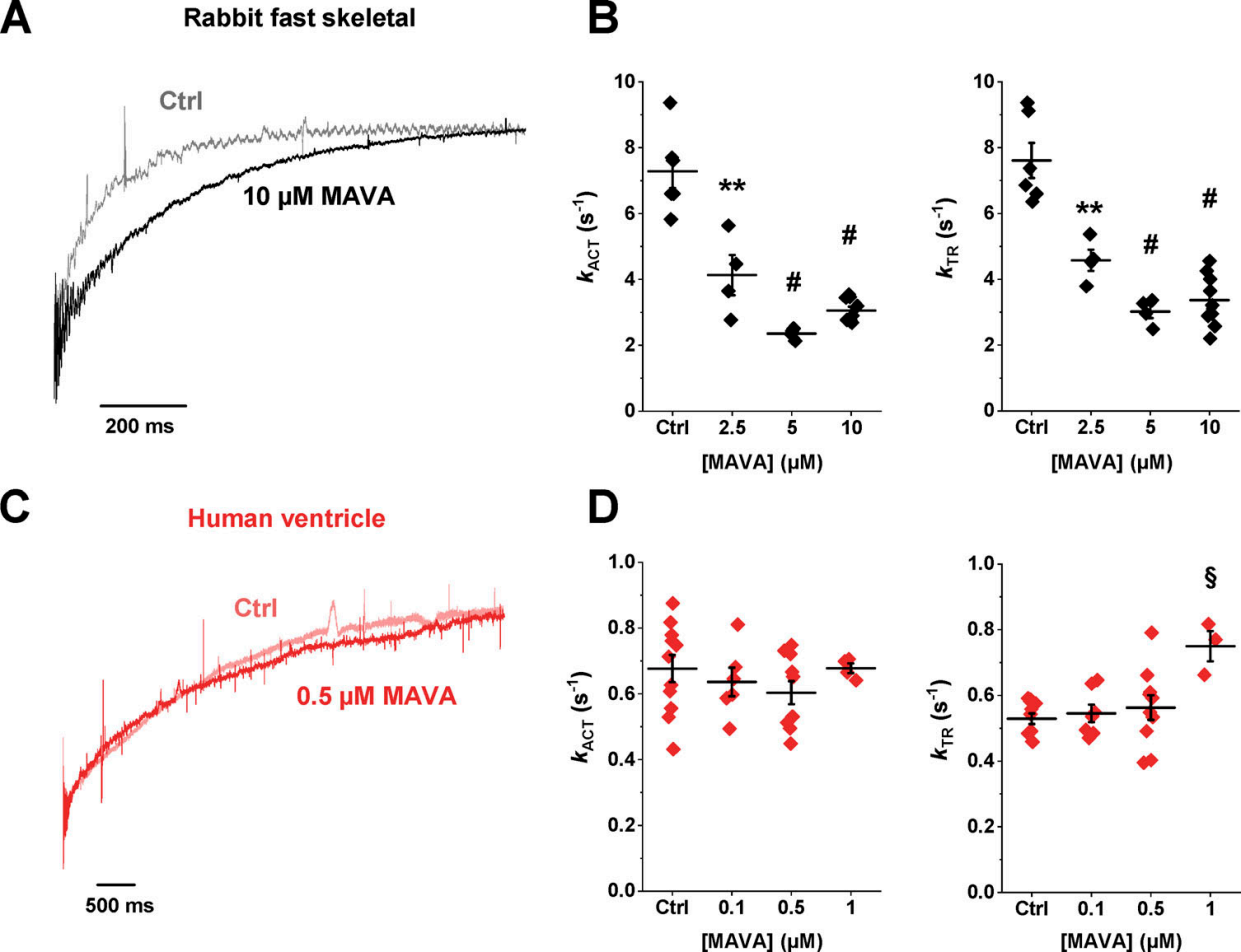
**MAVA differentially affects the kinetics of force development in rabbit fast skeletal and human ventricle myofibrils.** **(A)** Representative traces of force redevelopment (*k*_TR_) from a rabbit fast skeletal myofibril that was maximally activated to pCa 4.5 in the absence (Ctrl, gray) and presence (black) of 10 µM MAVA. **(B)** Mean *k*_ACT_ and *k*_TR_ values at different MAVA concentrations for rabbit psoas myofibrils. **(C)** Representative traces of force redevelopment from a human ventricular myofibril that was maximally activated to pCa 4.5 in the absence (Ctrl, light red) and presence (red) of 0.5 µM MAVA. **(D)** Mean *k*_ACT_ and *k*_TR_ values at different MAVA concentrations from human ventricular myofibrils. **, P < 0.005; #, P < 0.0001; §, P < 0.001 versus Ctrl. Error bars, ± SEM.

As shown in Fig. 3 and Table 1, the impact of MAVA on sarcomere kinetics was very different in fast skeletal and slow cardiac sarcomeres. In fast skeletal myofibrils, the kinetics of force development (both *k*_ACT_ and *k*_TR_) were strongly depressed by MAVA (Fig. 3, A and B), with a higher sensitivity compared with the depressant effect of the drug on tension (IC_50_ 2.51 ± 0.68 µM versus 7.00 ± 0.91 µM). This was clearly observed at 1 µM MAVA, a drug concentration that depressed *k*_TR_ by 36% but did not affect maximal Ca^2+^-activated tension (−2 ± 2%, *n* = 4). At variance with skeletal myofibrils, MAVA had no depressant effect on the kinetics of force generation of human ventricular myofibrils (Fig. 3, C and D). For MAVA doses higher than the IC_50_ for tension reduction, *k*_TR_ of human ventricular myofibrils was even significantly higher than that measured in the absence of the drug (see Fig. 3 D and Table 1).

Results from a set of experiments in rabbit psoas myofibrils showed that the inhibiting effect of MAVA on both force and *k*_TR_ was substantially unaffected by P_i_ in the 0–1 mM concentration range (Table 2), with only a slight increase in the effect on force and a slight decrease in the effect on *k*_TR_ at 10 µM MAVA and 1 mM P_i_ (P = 0.05, *n* = 4).

**Table 2.** Effect of MAVA on the mechanical properties of myofibrils from rabbit fast skeletal in the 0–1 mM [P_i_] range

Rabbit fast skeletal	P_MAVA_/P_0_	*k*_TR MAVA_/*k*_TR_	*k*_MAVA−_/*k*_TR_
**5 µM MAVA**			
0 P_i_	0.48 ± 0.05 (4)	0.35 ± 0.03 (4)	0.48 ± 0.07 (4)
Nominal P_i_	0.62 ± 0.02 (3)	0.24 ± 0.03 (3)	0.40 ± 0.06 (3)
1 mM P_i_	0.52 ± 0.02 (5)	0.36 ± 0.03 (5)	0.63 ± 0.07 (5)
**10 µM MAVA**			
0 P_i_	0.49 ± 0.05 (4)	0.39 ± 0.06 (4)	0.52 ± 0.05 (4)
Nominal P_i_	0.44 ± 0.06 (9)	0.30 ± 0.02 (5)	0.45 ± 0.04 (9)
1 mM P_i_	0.30 ± 0.03 (4)	0.56 ± 0.11 (4)	0.51 ± 0.05 (4)

Each group of data was collected from 5 and 10 µM MAVA-jump experiments in different rabbit fast skeletal myofibril batches in Ctrl conditions (Nominal P_i_ ∼170 μM) or the presence of increased (1 mM P_i_) or enzymatically reduced (0 P_i_) P_i_ concentration. All values are given as mean ± SEM; values in parentheses are the myofibril numbers. MAVA index refers to parameters measured in 5 or 10 µM MAVA, as indicated. The one way-ANOVA analysis revealed no systematic differences between each data set.

**Figure 4.**
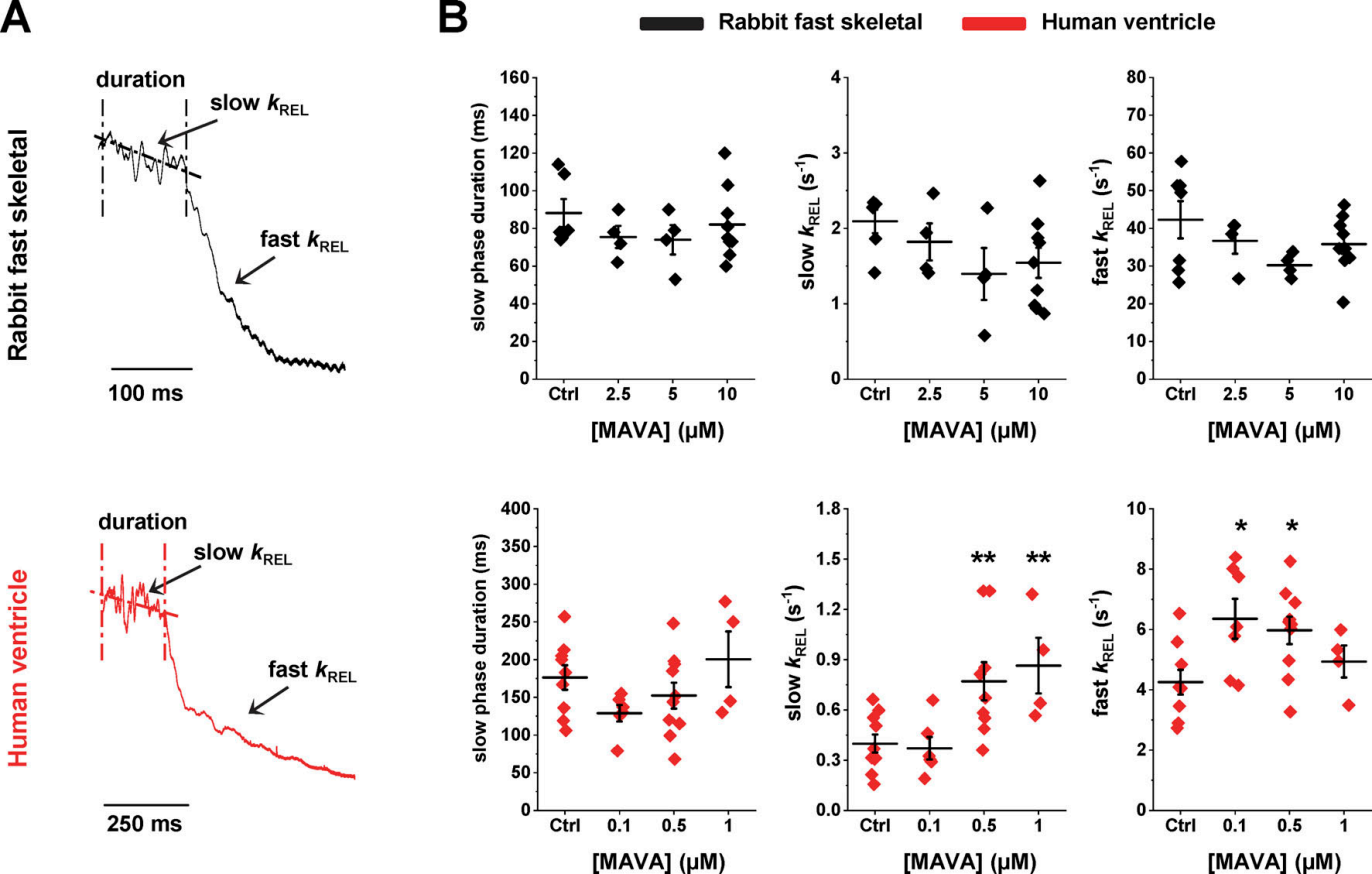
**MAVA differentially affects the kinetics of relaxation in rabbit fast skeletal and human ventricle myofibrils.** **(A)** Enlargements of the relaxation phase (from pCa 4.5 to pCa 9.0) from rabbit fast skeletal (top, black) and a human ventricular (bottom, red) myofibrils. As shown, full tension relaxation from maximal activation is biphasic in both myofibril systems. The rate constant of the early slow force decline (slow *k*_REL_) is estimated from the slope of the regression line fitted to the force trace normalized to the entire amplitude of the force relaxation transient. The rate constant for the final fast phase of tension decline (fast *k*_REL_) is estimated from monoexponential fit. **(B)** Mean values of slow phase duration, slow *k*_REL_ and fast *k*_REL_ at different MAVA concentrations for rabbit psoas (top) and human ventricular (bottom) myofibrils. **, P < 0.005; *, P < 0.05 versus Ctrl. Error bars, ± SEM.

As previously observed in both skeletal and human cardiac myofibrils (Tesi et al., 2002b; Piroddi et al., 2007), the time course of full force relaxation following Ca^2+^ removal below the contraction threshold (pCa 9.0; see Fig. 4 A) was biphasic, starting with a slow, seemingly linear phase followed (after a “shoulder”) by a fast, exponential, relaxation phase. The rate constant of the linear phase (slow *k*_REL_) and the rate constant of the exponential phase (fast *k*_REL_) were respectively significantly slower and faster than *k*_ACT_ or *k*_TR_ (Poggesi et al., 2005). In rabbit psoas myofibrils, MAVA had no significant effect on force relaxation; both the overall duration and the kinetics of the two phases of force decay were the same (Fig. 4 B, upper panels). On the contrary, in human ventricular myofibrils (Fig. 4 B, lower panels), MAVA significantly increased both slow *k*_REL_ and fast *k*_REL_, with an overall accelerating effect that accompanied the lack of effect on the kinetics of force generation (or even the increased *k*_TR_ observed at 1 µM MAVA; Table 1). As MAVA is known to act on multiple steps of myosin chemomechanical cycle (Kawas et al., 2017), the comparison of its effects on the kinetics of force development and relaxation in rabbit psoas and human ventricular myofibrils likely reflects and “senses” differences in the distribution of cross-bridge states along the cycle and in rate-limiting steps between fast and slow muscles (see Discussion).

### MAVA jump experiments prove that MAVA favors fast and fully reversible cross-bridge detachment

**Figure 5.**
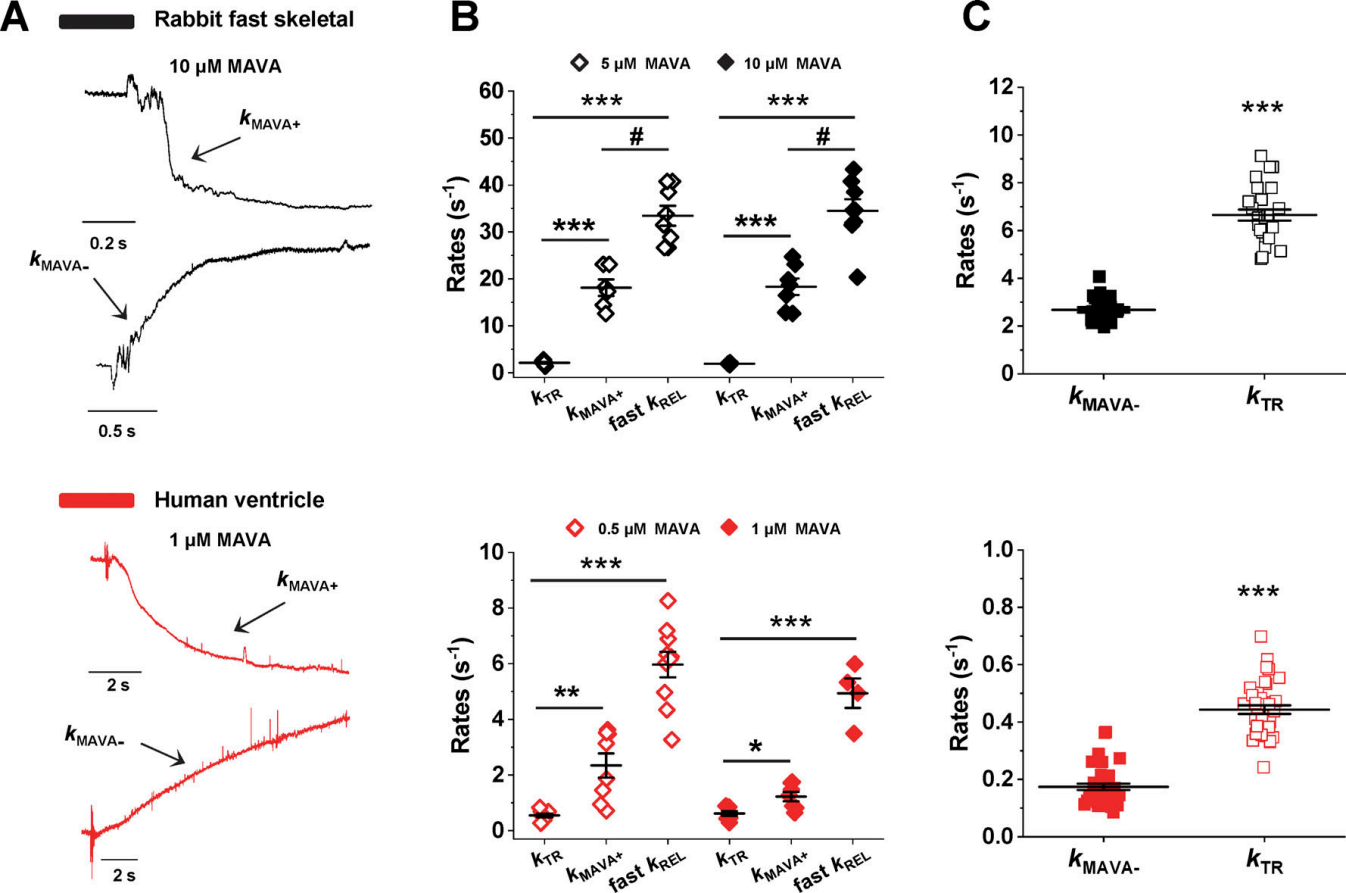
**The kinetics of MAVA jumps gives insight in the underlying mechanism of MAVA action.** **(A)** Enlargements of MAVA-jump traces in Fig. 1 B made to highlight the kinetics of force changes induced by MAVA addition (*k*_MAVA+_) and removal (*k*_MAVA__−_) in rabbit psoas (black) and human ventricle (red) myofibrils. **(B)** Mean data of *k*_TR_ and *k*_MAVA+_ obtained from MAVA jump protocols are compared with fast *k*_REL_ measured in activation–relaxation cycles at two concentrations of MAVA. [MAVA] for skeletal myofibrils: 5 µM (open symbols) and 10 µM (closed symbols). [MAVA] for human cardiac myofibrils: 0.5 µM (open symbols) and 1 µM (closed symbols). ***, P < 0.0001; **, P < 0.001; *, P < 0.01; #, P < 0.0005 between datasets as indicated by bars. **(C)** Mean data of *k*_MAVA−_ (closed symbols) at two different MAVA concentrations are compared with Ctrl *k*_TR_ (open symbols) for skeletal and cardiac myofibrils. ***, P < 0.0001. Error bars, ± SEM.

When subjected to MAVA jumps, both myofibril types responded with a fully reversible rapid relaxation-like force drop (the same traces reported in Fig. 1 B are shown enlarged in Fig. 5 A) whose kinetics approached the kinetics of the relaxation phase (Fig. 5 B).

For [MAVA] ≥ IC_50_, the drop in force following sudden exposure to the drug was biphasic in both fast skeletal and cardiac muscle myofibrils. As it was difficult to resolve the two phases of the drop in force, *k*_MAVA+_ was mostly estimated from the larger fast phase of the force decay transient. In both myofibril types, *k*_MAVA+_ at final [MAVA] ≥ IC_50_ was found significantly higher than *k*_TR_, and in the rabbit psoas, it attained a value of ∼50% of fast *k*_REL_ (Fig. 5 B). For [MAVA] well below IC_50_ (i.e., for force drops < 25%), the kinetics of the MAVA force drop became monophasic and approached the rate of the slow phase of force relaxation. This was clearly resolved in human ventricular myofibrils for 0.1 µM MAVA jumps (relative force, 0.80 ± 0.03; *n* = 8), where *k*_MAVA+_ and slow *k*_REL_ were found to be 0.42 ± 0.07 s^−1^ and 0.48 ± 0.13 s^−1^, respectively, and *k*_TR_ was 0.41 ± 0.05 s^−^^1^ (*n* = 7). The similarities among *k*_MAVA+_, *k*_REL_, and *k*_TR_ observed when the amount of residual tension following the tension drop is well above 50% were expected from the behavior of the kinetics of myofibril force relaxation transient following a reduction in [Ca^2+^] from maximal to submaximal activation levels (Tesi et al., 2000).

Interestingly, in both myofibril types, the kinetics of the increase in force following MAVA removal (*k*_MAVA_*_−_*) was significantly slower than *k*_TR_ in the absence of MAVA and fairly independent from the initial drug concentration or the initial/final force (Fig. 5 C). The mean value of the ratio of *k*_MAVA_*_−_* over *k*_TR_ was 0.41 ± 0.02 (*n* = 19) and 0.43 ± 0.05 (*n* = 23) in rabbit psoas and human ventricular myofibrils, respectively. In rabbit psoas myofibrils, this value was again unaffected by P_i_ in the 0–1 mM range (Table 2). The value of *k*_MAVA_*_−_* seemed then to be settled by a slower process that could be related either to the washout of the drug from the myofibril lattice or to MAVA inducing a relatively slow transition of detached heads to the SRX state as it had been previously suggested by in vitro ATPase measurements and structural studies (Green et al., 2016; Kawas et al., 2017; Anderson et al., 2018). Of note, the mean delay observed between the solution switch that suddenly removes MAVA and the start of the force increase was 708 ± 29 ms (*n* = 27), which is approximately three times longer than the delay observed between the sudden exposition to the drug and the start of the force decay (192 ± 9 ms; *n* = 26).

**Figure 6.**
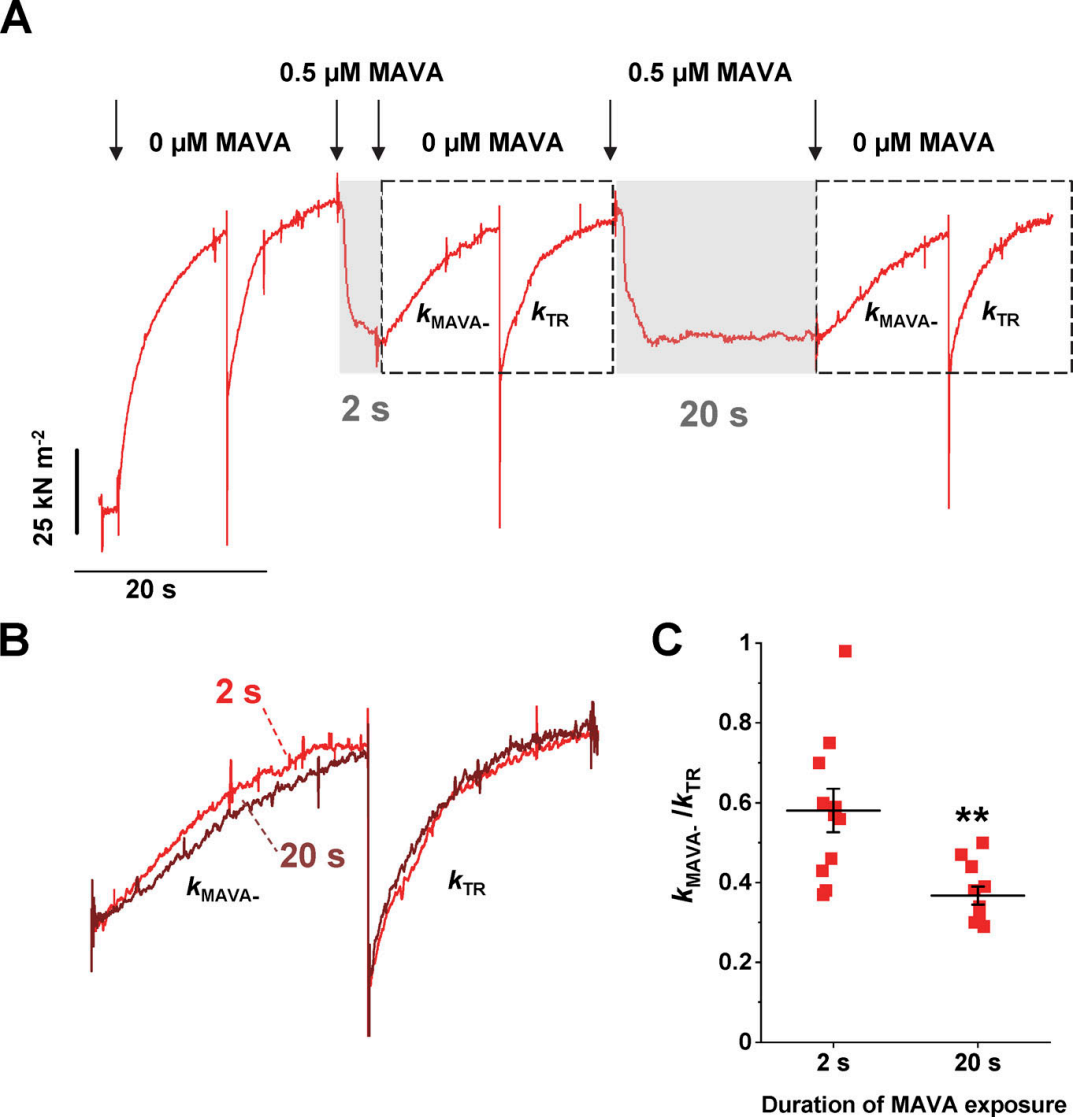
**Double MAVA-jump protocol suggests slower transitions of detached heads into “parked” states.** **(A)** Representative trace of a double MAVA jump protocol exposing a cardiac myofibril to 0.5 µM MAVA and back for 2 s and 20 s respectively. Arrows mark the time of indicated fast solution changes. **(B)** Traces showing force recover after 2 s (red) and 20 s (dark red) exposure to MAVA are shown superimposed. **(C)** Mean data of the ratio *k*_MAVA−_*/k*_TR_ for the two different MAVA exposure times. **, P < 0.005, 20-s versus 2-s exposure to MAVA. Error bars, ± SEM.

To further investigate this point, human ventricular myofibrils were tested with a double-jump protocol (Fig. 6 A). In the first jump, the myofibril was exposed to the drug for a relatively brief period (2 s) that just allowed force to drop to a steady state. After the complete force recovery that followed the sudden removal of the drug, the myofibril was again suddenly exposed to the same MAVA concentration, but for a longer duration (20 s) before the final sudden drug removal. Of note, 20 s was the drug exposure time used in MAVA jump protocols of the present study. Interestingly, the kinetics of the increase in force on MAVA removal was significantly faster for the short exposure time than for the longer exposure time (Fig. 6 B). At 2-s exposure time, the difference between the rate of force recovery on MAVA removal *k*_MAVA_*_−_* and the control values of *k*_TR_ was significantly reduced (Fig. 6 C). The result is not expected from the simple washout time of the drug from the lattice (that can only affect the delay between drug removal and start of force recovery) and may support the idea that MAVA favors the recruitment of detached cross-bridges to states not immediately available for force generation.

## Discussion

Mechanical measurements in myofibrils proved that MAVA inhibits maximal isometric tension with the same myosin isoform specificity (i.e., the same IC_50_) as previously found for the ATPase activity of the same myofibril systems (Kawas et al., 2017). Present results represent a significant advancement of knowledge on the impact of MAVA on human cardiac sarcomeres. What is known about MAVA effects in human myocardium is not much and has been obtained in freely shortening isolated paced iPSC-CMs compared with isogenic WT iPSC-CMs (Toepfer et al., 2020) or skinned ventricular strips (Anderson et al., 2018; Awinda et al., 2020). In these latter studies, the inhibitory action of MAVA on force was estimated at only one concentration of the drug (very high in the former study [50 µM] and approximately the IC_50_ in the latter one [0.5 µM]). Interestingly, myofibril experiments showed that MAVA was also very effective at inhibiting ADP-stimulated contraction in both fast skeletal and cardiac muscle (i.e., at inhibiting contractile force generated through Ca^2+^-independent activating mechanisms).

This result supports the conclusion of Tanner and co-workers (Awinda et al., 2020) who attributed the MAVA-dependent decrease of passive force of skinned human ventricular strips at 37°C to the decrease of a myosin-based contribution to thin-filament activation. MAVA, instead, did not affect the passive properties of skeletal and cardiac myofilaments in a wide range of sarcomere lengths, confirming previous observations about the lack of effect of 2,3-butanedione monoxime (BDM) on the sarcomere length–resting tension relation of isolated myofibrils (Scellini et al., 2017). This indicates that in the usual conditions of myofibril experiments (15°C, 5 mM ATP, presence of ATP regenerating system and no ADP), Ca^2+^-independent force generation is not significant, at variance with what may occur in the intact tissue. In multicellular cardiac preparations at physiological temperature and [Mg^2+^], a small but significant degree of Ca^2+^-independent tension is present (Sequeira et al., 2015) that may account for the reported inhibitory effect of MAVA on cardiac passive properties at 37°C (Awinda et al., 2020). This effect of the drug may be able to counteract the basal sarcomere activation in the virtual absence of Ca^2+^ that, together with the increase in myofilament Ca^2+^ sensitivity, often contributes to the diastolic dysfunction described in human HCM (Sequeira et al., 2015; Tardiff et al., 2015; Ferrantini et al., 2017).

One additional novelty of the present work is the characterization of the effects of MAVA on the kinetics of force development *k*_ACT_ or redevelopment *k*_TR_ in rabbit psoas and human ventricular myofibrils which disclosed a fundamental difference in the impact of the drug on fast and slow myosins. In the sarcomeres expressing fast myosin (*MYH1*), MAVA depressed the kinetics of force development (both *k*_ACT_ and *k*_TR_) in the same way as force, though with a slightly higher sensitivity. Unexpectedly, in human ventricular myofibrils expressing slow myosin (*MYH7*), MAVA did not depress the kinetics of force development; rather, at high doses, it increased them.

The effects observed in fast and slow myofibrils can be discussed in the light of the results of jump experiments showing that force almost instantaneously drops following the exposition to the drug in a relaxation-like fashion. This is consistent with MAVA inducing—in the first place—a fast shift of cross-bridges toward detached states. This behavior reminds the sudden drop in myofibril force following a jump increase in [P_i_] (Tesi et al., 2000; Stehle, 2017). The shift of cross-bridges toward detached states is expected, as MAVA has been shown to decrease the P_i_ release rate (Kawas et al., 2017; Anderson et al., 2018), an effect that is associated with the increase in the fraction of A−M.ADP.P_i_ cross-bridge states in rapid equilibrium with the detached A+M.ADP.P_i_ states. The decrease of the P_i_ release rate could also account for the decrease of *k*_ACT_ and *k*_TR_ observed in rabbit psoas myofibrils (Gordon et al., 2000), leaving yet unexplained the effect of MAVA in human ventricular myofibrils. The hypothesis that a fast binding of MAVA to its allosteric site on myosin head triggers its mechanical effect is strongly supported by the fact that the delay between the solution switch and the drop in force in MAVA jump experiments is not significantly different from the delays in the myofibril force responses following Ca^2+^ activation or removal.

**Figure 7.**
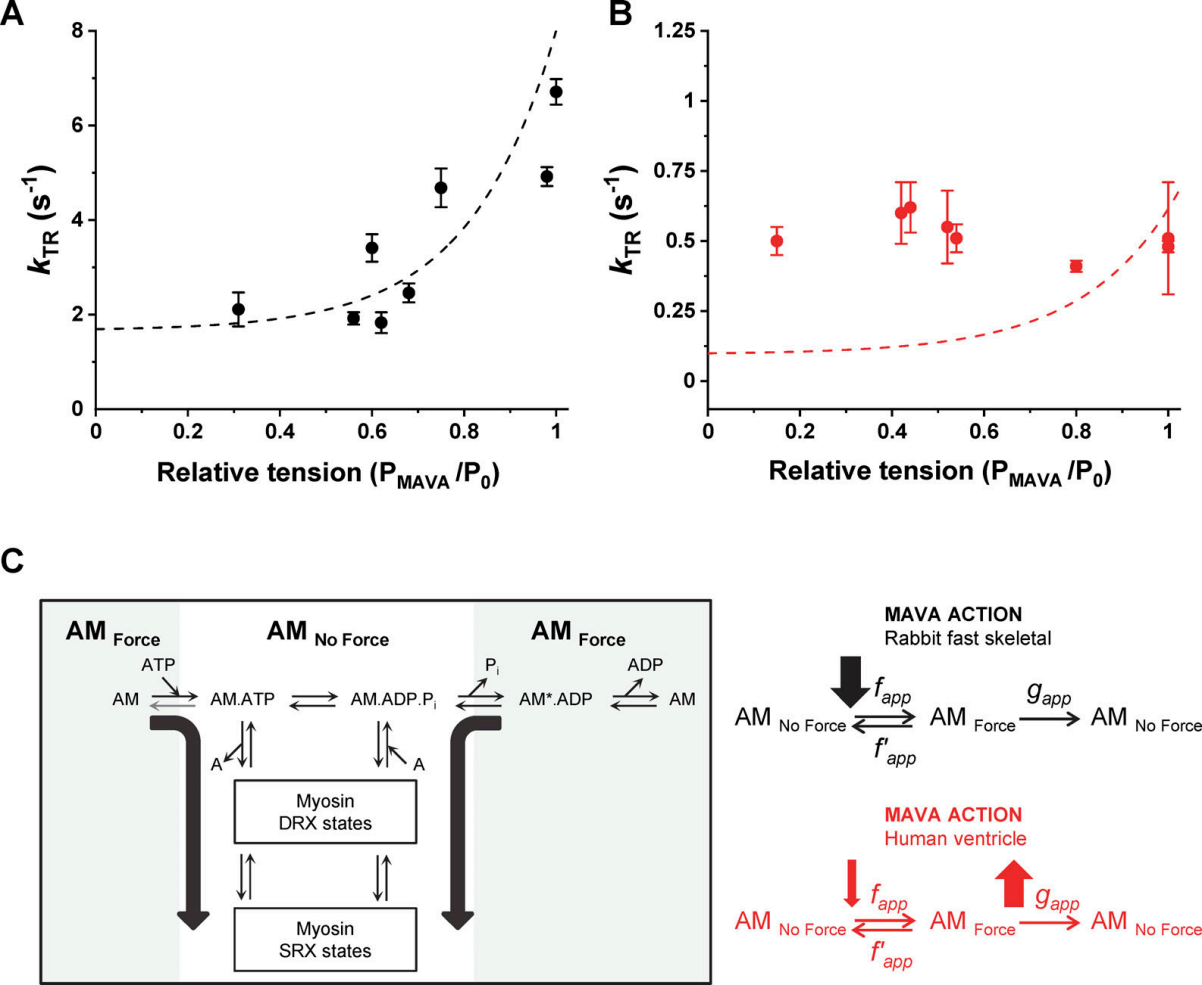
**The differential effect of MAVA on human ventricular and rabbit psoas myofibrils points to isoform-specific differences in the consequences of the binding of the ligand to the allosteric site.** **(A) ***k*_TR_ values in presence of MAVA from jump experiments in rabbit psoas myofibrils display the same dependence on force whether modulated by MAVA (experimental points) or Ca^2+^ activation level (dashed line; redrawn from Fig. 5 D in Tesi et al., 2000; 15°C). Isometric tension normalized for maximal Ctrl values from each jump protocol (data points are means ± SEM of four to eight myofibrils). **(B)** The same relation for human ventricle myofibrils (data points are means ± SEM of 4–20 myofibrils; dashed line redrawn from Fig. 3 D in Piroddi et al., 2007; 15°C). **(C)** Schematic of the mechanism of MAVA action in rabbit fast skeletal (black) and human ventricle (red) informed by data in this study. Two-state scheme for cross-bridge cycle incorporating DRX and SRX states (McNamara et al., 2015), with AM_No Force_ representing all weak binding states in rapid equilibrium with detached and AM_Force_ all strong and force-generating binding states. The transition from AM_No Force_ to AM_Force_ has an apparent rate constant ƒ_app_, whereas g_app_ describes the return to AM_No Force_ by means of ADP release and ATP binding. The apparent rate constant for the reverse transition ƒ′_app_ depends on [P_i_] and is small at nominal [P_i_] (∼170 µM).

In addition to the decrease in the rate of P_i_ release, the decrease in the kinetics of force development operated by MAVA in psoas myofibrils can be also explained by comparing the relations between the kinetics of force development and the level of force modulated by the drug or by the free [Ca^2+^] (Fig. 7 A). Several rather-accepted models of contraction regulation suggest that Ca^2+^ affects the kinetics of force development in an indirect way (i.e., by modulating the availability of actin regulatory units for the interaction with myosin; Brenner, 1988; Poggesi et al., 2005; Stehle et al., 2009; Campbell, 2014). By analogy, the decrease in the number (*N_a_*) of myosin heads functionally available for interacting with actin (at full Ca^2+^ activation) would cause a drop in force as force in the sarcomere is settled by the product of the intrinsic force per cross-bridge times the total number of functionally accessible actin-interacting heads *N_a_* and the duty ratio (Spudich, 1994). If Ca^2+^ is modulating *N_a_* on the thin-filament (actin) side, MAVA would do the same on the thick filament side and decrease *N_a_* by “sequestering” available DRX heads into the SRX state (Fig. 7 C), as extensively proved by biochemical and structural evidence collected over the last few years (Spudich, 2019). MAVA would then decrease the occupancy of the open actin state (McKillop and Geeves, 1993; Geeves and Lehrer, 1994), i.e., the probability that cross-bridges enter the force-generating cycle and then modulate the kinetics of force development in the same “apparent” way as Ca^2+^ is doing. Of course, the decrease in the number of cycling heads induced by MAVA, besides accounting for the force decrease, would also lower the absolute amount of P_i_ released in the unit of time and, therefore, the observed steady-state ATPase, as experimentally observed (Green et al., 2016; Kawas et al., 2017).

In terms of a two-state model of the cross-bridge cycle (Fig. 7 C; Huxley, 1957; Brenner, 1988; Gordon et al., 2000), MAVA would, therefore, operate an indirect rate modulation of *f_app_* in the same way as Ca^2+^ is doing but through a ligand based modulation of heads “ready to attach.” At high levels of activation, assuming a fast Ca^2+^ switch of regulatory units, the rate of force development that reflects the cross-bridge turnover rate (*f_app_* + *g_app_*) will be mostly set by *f_app_* if *f_app_* >> *g_app_* (Brenner, 1988; Ferrantini et al., 2009). Results from rabbit psoas myofibrils fully support this interpretation showing that the relation between *k*_ACT_ or *k*_TR_ and tension is exactly the same whether tension is modulated by MAVA (Fig. 7 A, experimental dot points) or Ca^2+^ (Fig. 7 A, dashed line; Tesi et al., 2002b).

Consistent with this interpretation is the effect of MAVA on the relaxation phase of rabbit psoas myofibrils. In fast muscle, MAVA does not significantly affect overall force relaxation and leaving both slow and fast *k*_REL_ unchanged. This suggests no major effect of the drug on the apparent rate of cross-bridge detachment (*g_app_*) in rabbit psoas myofibrils. This conclusion is in keeping with previous findings showing slow *k*_REL_ (which reflects *g_app_*; Huxley, 1957; Poggesi et al., 2005; Vitale et al., 2021) to be independent of *N_a_* also in the case of Ca^2+^ modulation and at the same time to be very sensitive to [P_i_] (Tesi et al., 2002b; Poggesi et al., 2005).

Several arguments could be considered when interpreting present results in human ventricular myofibrils. First of all it is important to notice that in human ventricular myofibrils, the relation between the kinetics of force generation and tension (modulated by Ca^2+^) is rather flat compared with that of rabbit psoas myofibrils (Piroddi et al., 2007), while individual rates are low and scattered (Witjas-Paalberends et al., 2013; Piroddi et al., 2019). Similar results have been previously obtained in ventricular trabeculae from animal models reporting flat- (Hancock et al., 1996) or low-slope linear (Wolff et al., 1995) relationships for the dependence of force kinetics on activating Ca^2+^ concentration and isometric force. Here, *k*_TR_ data from MAVA jump experiments produced a clearly flat relation between the kinetics of force generation and tension as modulated by MAVA (see Fig. 7 B). This suggests that under our experimental conditions (15°C), the kinetics of force generation of human ventricular myofibrils is less coupled to or even uncoupled from actin states occupancy and is mostly settled by intrinsic cross-bridge cycling rates (centered on P_i_ release) at all force levels. This result disagrees with the inhibitory effect of MAVA previously inferred from indirect estimates of the rate of force generation from stretch activation protocols in mice trabeculae at 25°C (Mamidi et al., 2018) as well as from viscoelastic stiffness measurements in skinned human ventricular strips at 37°C (Awinda et al., 2020). These differences could result from the high temperature sensitivity of the P_i_-release step (White et al., 1997) and of the overall cross-bridge kinetics. As myofibril experiments cannot be performed at high temperature due to exacerbated mechanical rundown, experiments are planned in human cardiac trabeculae at physiological temperature to address this crucial point.

Interestingly, in human ventricular myofibrils, MAVA, at drug concentrations around IC_50_, induced a significant increase in slow *k*_REL_ and then on the apparent rate of cross-bridge detachment (*g_app_*; Fig. 7 C). This direct kinetic effect in the presence of MAVA may be due to alterations of the ADP release steps (Stehle et al., 2009; Stehle and Iorga, 2010; Walklate et al., 2016), as already suggested in human ventricular strips from measurement of viscoelastic stiffness (Awinda et al., 2020).

The differential effect of MAVA on human ventricular and rabbit psoas myofibrils points to isoform-specific differences associated with the different occupancy of intermediates along the cycle (Mijailovich et al., 2017) or structural differences in the specific MAVA-binding sites to fast versus slow myosin heads, similarly to recently reported findings on omecamtiv mecarbil binding (Planelles-Herrero et al., 2017). However, the allosteric site of MAVA has not been yet described by structural studies, leaving the question open for future research.

As to the widely reported effect of MAVA to shift myosin heads from DRX to SRX states, a mechanical study, like the present one, can only provide indirect evidence, as both DRX and SRX are detached states that cannot be mechanically probed. The indirect evidence reported here is the much slower rate of the force rise following MAVA removal *k*_MAVA_*_−_* compared with *k*_TR_ and its dependence on the time of exposure to the drug in the double-jump experiments (Fig. 6 C). Interestingly, jump experiments in fast and slow myofibrils using BDM as myosin inhibitor always produced force transients of kinetics not significantly different from *k_TR_* measured in the presence or absence of the ligand (Tesi et al., 2002a). Future experiments combining structural and mechanical approaches are needed to functionally investigate the mechanism of action MAVA and other myosin inhibitors in relation to the states of detached motor heads on thick filament.

In conclusion, the present results contribute to the understanding of the mechanism of action of MAVA in striated muscle. The effect of the drug is primarily associated with a decrease in the number of heads available for interaction with actin. In view of the currently mostly accepted hypothesis that explains the hypercontractile phenotype associated with HCM with an increase in the number of available heads (Spudich, 2019), MAVA then seems to represent one ideal therapeutic intervention for HCM patients at any stage of the disease. The acceleration of force relaxation specifically observed in human cardiac myofibrils following exposure to MAVA and the inhibition of the ADP-stimulated force developed in the virtual absence of Ca^2+^ provide additional value to the use of this drug in HCM patients. In addition, the expected normalization of myofilament Ca^2+^ sensitivity in the presence of MAVA could reduce arrhythmia susceptibility in this potentially life-threatening disease. Together with the documented rescue of HCM in animal models following MAVA treatment (Green et al., 2016; Stern et al., 2016; Mamidi et al., 2018; Toepfer et al., 2019) and the growing body of positive results in MAVA clinical studies (Heitner et al., 2019; Fumagalli et al., 2020; Olivotto et al., 2020), the present results increase the perspectives of future research of novel myofilament targeting drugs for cardiac as well as skeletal myopathies.
